# Changes in Blood Lactate Levels after a 40-km Endurance March Depend on Fluid Replacements: A Case Study on Korean Military Academy Cadets

**Published:** 2017-02

**Authors:** Jae-Woo KIM, Wi-Young SO, Kwang Suk CHA

**Affiliations:** 1. Dept. of Physical Education, Korea Military Academy, Seoul, Korea; 2. Sports and Health Care Major, Korea National University of Transportation, Chungju-si, Korea; 3. Division of Sport Sciences, Konkuk University, Chungju-si, Korea

## Dear Editor-in-Chief

For soldiers, surviving the war, increasing their mission capability rates, and physical fitness levels such as endurance, strength, mobility, and flexibility are important (1). In particular, endurance is an important physical fitness factor essential while soldiers perform their missions (1). Blood lactate threshold (LT) is an excellent predictor of an individual’s endurance capacity (2); that is, individuals with high fitness levels have high LTs.

Water constitutes approximately 60%–70% of the total body weight, 90% of blood volume, and 85% of the brain in the living systems (3). Fluid replacements with water or sports drink (electrolyte beverage) during/after exercise training can effect performance enhancement (4–5). Nevertheless, it is unclear whether blood lactate levels change with fluid replacements after a 40-km endurance march. Therefore, the purpose of this study was to examine changes and recovery patterns of LTs depending on fluid replacements in Korean military academy cadets after a 40-km endurance march.

Eighteen male cadets enrolled in the Korea Military Academy, Seoul, Republic of Korea, were included as participants in 2015. They were randomly assigned to the water group (n=8) and sports drink group (n=8). Their average age (± standard deviation) was 19.22 ± 0.88 yr; average height, 174.53 ± 5.07 cm; and average weight, 67.22 ± 4.56 kg; and average body mass index, 22.11 ± 1.82 kg/m^2^.

All study participants provided informed consent, and the study was approved by Korea Military Academy.

They did not exercise regularly and had no health problems. All participants submitted a written consent form for participating in the study. LTs were obtained using the blood lactate test meter (Lactate Pro™ LT-1710; Arkray Inc., Japan) immediately after the 40-km endurance march, and 15, 30, 45, and 60 min after the march. Water and sports drink (6% carbohydrate; 20.9 mEq/L Na+; 6.1 mEq/L K+: and 9.5 mEq/L CI−) solutions were prepared at 15 °C, the temperature at which absorption rate is the highest and that brings a sense of refreshment (6). In this study, we used Mann Whitney U-test, a non-parametric method, because the number of participants in each group was less than 30, and the data would not allow a normal distribution; moreover, such data may not be reliable according to the central limit theorem (7). Statistical significance was set at *P*<0.05 using SPSS Window version 18.0 (Chicago, IL, USA).

Changes in recovery of LTs after 40-km endurance march depends on fluid replacements in Korea Military Academy cadets are shown in Table 1.

**Table 1:** Differences in recovery patterns of blood lactate levels in Korea Military Academy cadets after a 40-km endurance march depending on fluid replacements

**Lactate level**	**Water (n= 9)**	**Sports drink (n= 9)**	***z***	***P***
Immediately after the 40-km endurance march	4.34 ± 2.34	3.01 ± 1.39	−1.370	0.182
After 15 min	4.39 ± 2.62	3.60 ± 2.80	−1.595	0.118
After 30 min	3.40 ± 1.11	3.82 ± 1.22	−0.974	0.351
After 45 min	2.91 ± 1.36	4.19 ± 1.23	−2.211	0.026^*^
After 60 min	4.18 ± 0.92	3.56 ± 0.92	−1.289	0.211

Data are presented as mean ± standard deviation (Unit: mmol/L)

* *P*<0.05 as determined using the Mann Whitney *U*-test

There were no statistically significant differences in LTs between the water and sports drink groups immediately after 40-km endurance march (*P*=0.182), and 15 (*P*=0.118), 30 (*P*=0.351), and 60 (*P*=0.211) min after the march. However, statistically significant differences in LTs were noted only 45 min after the march (*P*=0.026). Although, only the difference in post-45 min lactate level was statistically significant between the two groups, the recovery pattern of LTs of Korea Military Academy cadets after the march was not statistically significant between water and sports drink groups.
